# Supplementary material for: COVID-19 and malaria co-infection: a systematic review of clinical outcomes in endemic areas
Source: PeerJ. 2024 Apr 18;12:e17160. doi: 10.7717/peerj.17160 (PMC11032658; doi:10.7717/peerj.17160)
Supplement: Supplemental Information 2 [file peerj-12-17160-s002.docx]

**JBI Checklist for Case reports**

| JBI checklist questions | (Huang *et al.*, 2022) | (Boonyarangka et al., 2022) | (Lee, Hong and Kim, 2022) | (Asmarawati *et al.*, 2022) | (SUAREZ *et al.*, 2022) | (Scalisi *et al.*, 2022) | (Shahid *et al.*, 2021) | (Jochum *et al.*, 2021) | (Caglar *et al.*, 2021) | (Pusparani, Henrina and Cahyadi, 2021) | (Indari *et al.*, 2021) | (Chen et al., 2021) | (Kishore *et al.*, 2020) |
| --- | --- | --- | --- | --- | --- | --- | --- | --- | --- | --- | --- | --- | --- |
| 1.       Were patient’s demographic characteristics clearly described? | No | Yes | Yes | Yes | No | Yes | Yes | Yes | Yes | Yes | Yes | Yes | Yes |
| 2.       Was the patient’s history clearly described and presented as a timeline? | Yes | Yes | Yes | Yes | No | Yes | Yes | Yes | Yes | Yes | Yes | No | Yes |
| 3.       Was the current clinical condition of the patient on presentation clearly described? | Yes | Yes | Yes | Yes | Unclear | Yes | Yes | Yes | Yes | Yes | Yes | Yes | Yes |
| 4.       Were diagnostic tests or assessment methods and the results clearly described? | Yes | Yes | Yes | Yes | Yes | Yes | Yes | Yes | Yes | Yes | Yes | Yes | Yes |
| 5.       Was the intervention(s) or treatment procedure(s) clearly described? | Yes | Yes | Yes | Yes | Yes | Yes | Yes | Yes | Yes | No | Yes | Yes | Yes |
| 6.       Was the post-intervention clinical condition clearly described? | Yes | Yes | Yes | Yes | Yes | Yes | Yes | Unclear | Yes | Unclear | Yes | No | No |
| 7.       Were adverse events (harms) or unanticipated events identified and described? | Unclear | Yes | Unclear | Unclear | No | Yes | Yes | No | Yes | Unclear | Yes | Unclear | No |
| 8.       Does the case report provide takeaway lessons? | Yes | Yes | Yes | Yes | Yes | Yes | Yes | Yes | Yes | Yes | Yes | Yes | Yes |

**JBI Checklist for Cohort studies**

| JBI checklist questions | (Achan et al., 2022) | (Hussein et al., 2022) | Muyinda *et al.*, 2022) | (Abbasher Hussien Mohamed Ahmed *et al.*, 2022) | ((Mahajan *etal*., 2020) |
| --- | --- | --- | --- | --- | --- |
| 1. Were the two groups similar and recruited from the same population? | Yes | Yes | Yes | Yes | Yes |
| 1. Were the exposures measured similarly to assign people to both exposed and unexposed groups? | Yes | Yes | Yes | Yes | No |
| 1. Was the exposure measured in a valid and reliable way? | Yes | Yes | Yes | Yes | Yes |
| 1. Were confounding factors identified? | Yes | Yes | No | No | No |
| 1. Were strategies to deal with confounding factors stated? | No | Yes | No | No | Yes |
| 1. Were the groups/participants free of the outcome at the start of the study (or at the moment of exposure)? | Unclear | No | Unclear | Yes | Unclear |
| 1. Were the outcomes measured in a valid and reliable way? | Yes | Yes | Yes | Yes | Yes |
| 1. Was the follow up time reported and sufficient to be long enough for outcomes to occur? | Yes | Yes | Yes | Unclear | Unclear |
| 1. Was follow up complete, and if not, were the reasons to loss to follow up described and explored? | Yes | Yes | No | Unclear | Unclear |
| 1. Were strategies to address incomplete follow up utilized? | No | No | No | No | No |
| 1. Was appropriate statistical analysis used? | Yes | Yes | Yes | Yes | Yes |

**JBI Checklist for case series**

| JBI checklist questions | Were there clear criteria for inclusion in the case series? | Was the condition measured in a standard, reliable way for all participants included in the case series? | Were valid methods used for identification of the condition for all participants included in the case series? | Did the case series have consecutive inclusion of participants? | Did the case series have complete inclusion of participants? | Was there clear reporting of the demographics of the participants in the study? | Was there clear reporting of clinical information of the participants? | Were the outcomes or follow up results of cases clearly reported? | Was there clear reporting of the presenting site(s)/clinic(s) demographic information? | Was statistical analysis appropriate? |
| --- | --- | --- | --- | --- | --- | --- | --- | --- | --- | --- |
| Forero-Peña et al., 2022) | **Yes** | **No** | **Yes** | **Unclear** | **Yes** | **Yes** | **No** | **Unclear** | **No** | **Yes** |

**JBI Critical Appraisal Checklist for case reports**

Reviewer ______________________________________ Date_______________________________

Author_______________________________________ Year_________ Record Number_________

|  | Yes | No | Unclear | Not applicable |
| --- | --- | --- | --- | --- |
| 1. Were patient’s demographic characteristics clearly described? | □ | □ | □ | □ |
| 1. Was the patient’s history clearly described and presented as a timeline? | □ | □ | □ | □ |
| 1. Was the current clinical condition of the patient on presentation clearly described? | □ | □ | □ | □ |
| 1. Were diagnostic tests or assessment methods and the results clearly described? | □ | □ | □ | □ |
| 1. Was the intervention(s) or treatment procedure(s) clearly described? | □ | □ | □ | □ |
| 1. Was the post-intervention clinical condition clearly described? | □ | □ | □ | □ |
| 1. Were adverse events (harms) or unanticipated events identified and described? | □ | □ | □ | □ |
| 1. Does the case report provide takeaway lessons? | □ | □ | □ | □ |

Overall appraisal: Include □ Exclude □ Seek further info □

Comments (Including reason for exclusion)

________________________________________________________________________________________________________________________________________________________________________________________________________________________________________________________________________________________________

**JBI Critical Appraisal Checklist for cohort studies**

Reviewer ______________________________________ Date_______________________________

Author_______________________________________ Year_________ Record Number_________

|  | Yes | No | Unclear | Not applicable |
| --- | --- | --- | --- | --- |
| 1. Were the two groups similar and recruited from the same population? | □ | □ | □ | □ |
| 1. Were the exposures measured similarly to assign people to both exposed and unexposed groups? | □ | □ | □ | □ |
| 1. Was the exposure measured in a valid and reliable way? | □ | □ | □ | □ |
| 1. Were confounding factors identified? | □ | □ | □ | □ |
| 1. Were strategies to deal with confounding factors stated? | □ | □ | □ | □ |
| 1. Were the groups/participants free of the outcome at the start of the study (or at the moment of exposure)? | □ | □ | □ | □ |
| 1. Were the outcomes measured in a valid and reliable way? | □ | □ | □ | □ |
| 1. Was the follow up time reported and sufficient to be long enough for outcomes to occur? | □ | □ | □ | □ |
| 1. Was follow up complete, and if not, were the reasons to loss to follow up described and explored? | □ | □ | □ | □ |
| 1. Were strategies to address incomplete follow up utilized? | □ | □ | □ | □ |
| 1. Was appropriate statistical analysis used? | □ | □ | □ | □ |

Overall appraisal: Include □ Exclude □ Seek further info □

Comments (Including reason for exclusion)

________________________________________________________________________________________________________________________________________________________________________________________________

**JBI Critical Appraisal Checklist for Case Series**

|  | Yes | No | Unclear | Not applicable |
| --- | --- | --- | --- | --- |
| Were there clear criteria for inclusion in the case series? | □ | □ | □ | □ |
| Was the condition measured in a standard, reliable way for all participants included in the case series? | □ | □ | □ | □ |
| Were valid methods used for identification of the condition for all participants included in the case series? | □ | □ | □ | □ |
| Did the case series have consecutive inclusion of participants? | □ | □ | □ | □ |
| Did the case series have complete inclusion of participants? | □ | □ | □ | □ |
| Was there clear reporting of the demographics of the participants in the study? | □ | □ | □ | □ |
| Was there clear reporting of clinical information of the participants? | □ | □ | □ | □ |
| Were the outcomes or follow up results of cases clearly reported? | □ | □ | □ | □ |
| Was there clear reporting of the presenting site(s)/clinic(s) demographic information? | □ | □ | □ | □ |
| Was statistical analysis appropriate? | □ | □ | □ | □ |
