# Supplementary material for: COVID-19 and malaria co-infection: a systematic review of clinical outcomes in endemic areas
Source: PeerJ. 2024 Apr 18;12:e17160. doi: 10.7717/peerj.17160 (PMC11032658; doi:10.7717/peerj.17160)
Supplement: Supplemental Information 3 [file peerj-12-17160-s003.docx]

# The rationale for conducting the systematic review / meta-analysis

The rationale for conducting the systematic review is to address the potential impact of co-infection between COVID-19 and malaria, especially in areas where malaria is endemic. COVID-19 and malaria are significant global public health concerns, and their co-infection could potentially worsen the clinical outcomes and burden on healthcare systems, making it crucial to understand their interplay.

# The contribution that it makes to knowledge in light of previously published related reports, including other meta-analyses and systematic reviews

This systematic review aims to fill the existing evidence gap and contribute to knowledge in light of previously published reports, including other meta-analyses and systematic reviews. While there have been individual case reports studies and reviews on COVID-19 and malaria infection, a comprehensive, systematic review/meta-analysis analysis on the coinfection of COVID-19 and malaria is lacking. Understanding the relationship between COVID-19 and malaria co-infection in regions where malaria is endemic is of significant public health importance. It can offer valuable insights into the possible interactions between these diseases and how their geographic overlap may impact the prevalence and clinical presentation of COVID-19. Moreover, the review aims to identify potential risk factors and co-morbidities associated with co-infection, helping to guide targeted interventions and improve clinical management. This systematic review can provide a more objective and generalized assessment of the topic by addressing the limitations of individual case reports or previous reviews, in which none discussed this topic. It fills the knowledge gap by providing comprehensive insights into co-infection clinical outcomes and epidemiological implications. The findings of this study may have a significant impact on public health strategies, especially in regions where malaria is endemic, as they can inform policymakers and healthcare providers about the need for integrated approaches to address co-infection and mitigate its impact on healthcare systems.
